# Development of pathological reconstructed high‐resolution images using artificial intelligence based on whole slide image

**DOI:** 10.1002/mco2.39

**Published:** 2020-11-19

**Authors:** Yang Deng, Min Feng, Yong Jiang, Yanyan Zhou, Hangyu Qin, Fei Xiang, Yizhe Wang, Hong Bu, Ji Bao

**Affiliations:** ^1^ Laboratory of Pathology Key Laboratory of Transplant Engineering and Immunology NHC, West China Hospital Sichuan University Chengdu China; ^2^ Department of Pathology West China Second University Hospital Sichuan University China & key Laboratory of Birth Defects and Related Diseases of Women and Children (Sichuan University) Ministry of Education Chengdu China; ^3^ Department of Pathology West China Hospital Sichuan University Chengdu China; ^4^ Chengdu Knowledge Vision Science and Technology Co., Ltd. Chengdu China; ^5^ Frontiers Science Center for Disease‐related Molecular Network West China Hospital Sichuan University Chengdu China

**Keywords:** adult granulosa cell tumor, artificial intelligence, reconstructed high‐resolution, uterine leiomyosarcoma, whole slide image

## Abstract

Pathology plays a very important role in cancer diagnosis. The rapid development of digital pathology (DP) based on whole slide image (WSI) has led to many improvements in computer‐assisted diagnosis by artificial intelligence. The common digitization strategy is to scan the pathology slice with 20× or 40× objective, and the 40× objective requires excessive storage space and transmission time, which are significant negative factors in the popularization of DP. In this article, we present a novel reconstructed high‐resolution (HR) process based on deep learning to switch 20 × WSI to 40 × without the loss of whole and local features. Furthermore, we collected the WSI data of 100 uterine leiomyosarcomas and 100 adult granulosa cell tumors to test our reconstructed HR process. We tested the reconstructed HR WSI by the peak signal‐to‐noise ratio, structural similarity, and the blind/reject image spatial quality evaluator, which were 42.03, 0.99, and 49.22, respectively. Subsequently, we confirmed the consistency between the actual and our reconstructed HR images. The testing results indicate that the reconstructed HR imaging is a reliable method for the digital slides of a variety of tumors and can be available on a large scale in clinical pathology as an innovative technique.

## Graphical abstract

The × 40 objective requires excessive storage space and transmission time, which are significant negative factors in the popularization of digital pathology. In this article, we present a novel reconstructed high‐resolution process based on deep learning to switch × 20 WSI to × 40 without the loss of whole and local features. The testing results indicate that the reconstructed high‐resolution imaging is a reliable method for the digital slides of a variety of tumors, and can be available on a large scale in clinical pathology as an innovative technique.

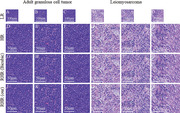

**Abbreviations**
AGCT: adult granulosa cell tumor
AI: artificial intelligence
CNNs: convolutional neural networks
DP: digital pathology
EDSR: enhanced deep super‐resolution network
HR: high‐resolution
LR: low‐resolution
PSNR: peak signal‐to‐noise ratio
RHR: reconstructed high‐resolution
SISR: single image super‐resolution
SRCNN: super‐resolution convolutional neural network
SSIM: structural similarity
WSI: whole slide image

## 1 INTRODUCTION

Pathological analysis is an important approach to diagnose diseases, particularly malignant tumors. The whole slide image (WSI), which meets the desired characteristics of high‐definition, high‐speed, and high‐throughput screening, provides the possibility for the digitization of traditional pathology slices, which lays a solid foundation for the development and application of digital pathology (DP).^1^, ^2^ With the development of DP, the storage and transmission of pathological images is now easier, which has led to many improvements in paleopathological consultation, digital management, and computer‐assisted diagnosis by artificial intelligence (AI). There have been more than 1 000 000 cases of paleopathological consultation in China over the last 10 years.^3^

In DP, the common digitization strategy is to scan the pathology slice with 20× or 40× objective. The data generated by the scan are called the WSI (or digital slide). Usually, the WSI of 40× is four times larger than that of 20× from the same slice, and hence, the storage space and transmission time of the data are four times. These increased costs are a significant negative factor in the popularization of DP. Although many lesions can be diagnosed at low or medium magnification, in some cases, even experienced pathologists need to observe cells and cell nuclear morphology at high magnification to further confirm the diagnosis. For example, dusty cytoplasms are often characterized by small cell neuroendocrine carcinoma. The longitudinal nucleus has a considerable role in the diagnosis of Langerhans histiocytosis and ovarian granulosa cell tumor. The observable mitotic has a good predictive effect on the diagnosis and differential diagnosis of uterine leiomyosarcoma. To provide clear and identifiable evidence for the diagnosis of tumors, whether it is an optical or a digital image at a high magnification ( 40×), it is necessary to adhere to the stringent requirements of the pathologist to observe the details in the nucleus. In this article, we present a novel reconstructed high‐resolution (RHR) process that can be used for WSI through deep learning.

For RHR imaging, deep learning techniques were recently developed for CT and MRI with great success.^4^ A few preliminary attempts have been made with a 3D convolutional neural network (CNN), generative adversarial network, and densely connected network.^5^, ^6^, ^7^ Thus, the RHR techniques have been used and have shown superb performance in imaging,^5^ and we expect the same in DP.

## 2 MATERIALS AND METHODS

### 2.1 The material of the pathological slides

In the present study, we used adult granulosa cell tumor (AGCT) of the ovary and uterine leiomyosarcoma as two application scenarios to test our resolution process. AGCT is a low‐grade malignant neoplasm with a significant propensity for late recurrence and metastasis.^8^ Histologically, GCTs are divided into adult and juvenile, with the former accounting for 95% of all GCTs.^8^, ^9^ Although some clinical manifestations such as estrogen stimulation and related hormone levels have an auxiliary role in the diagnosis of this tumor, the final diagnosis still depends on the traditional histopathological examination under light microscopy. Microscopically, tumor cells are arranged in trabecular, island‐like, pseudo‐adenoid, vesicular, or solid lamellae. Frequently, variable histological features and forms are mixed in the same tumor, which undoubtedly causes difficulties in the diagnosis of AGCT. A large number of studies have shown that the Call‐Exnar body and coffee bean‐like nucleus or longitudinal nucleus are the characteristic changes in typical AGCT, which is an important suggestion for the diagnosis of ovarian adult granulosa cells.^9^ Thus, for a more accurate diagnosis, it is necessary for pathologists to observe the details or features of the nucleus in × 40 magnification images.

Uterine leiomyosarcoma is the most common uterine sarcoma with a high malignancy and poor prognosis. According to the diagnostic criteria for histopathology developed by Bell et al,^10^ these are classified as (a) moderate diffuse atypia of tumor cells with observable necrosis; (b) moderate diffuse atypia of tumor cells without observable necrosis, mitotic figures ≥10/10 high‐power field (HPF); and (c) mild atypia of tumor cells with observable necrosis, mitotic figures are ≥10/10 HPF. The histological diagnosis of uterine leiomyosarcoma requires careful determination of three factors viz. coagulative necrosis, cell atypia, and the mitotic index. The mitotic index requires the pathologist to count at least four groups of ten HPFs in regions where mitotic activity is active. Therefore, clear and HR 40× magnification images are a basic prerequisite for pathologists to distinguish the mitotic figures from apoptotic cells, denatured cell nuclei, and nuclear debris.

We selected 45 cases of ovarian AGCT and 32 cases of uterine leiomyosarcoma diagnosed from the Department of Pathology, West China Second University Hospital, Sichuan University. All patients had no history of cancer and did not receive radiotherapy before surgery. The specimens were fixed with 4% neutral formaldehyde and subjected to conventional paraffin‐embedding, 4 μm thick sections, Hematoxylin and Eosin (HE) staining, and light microscopy examination. All HE slides were reviewed by two senior pathologists. Finally, a total of 100 HE slides from 45 cases of ovarian AGCT and 100 HE slides from 32 cases of uterine leiomyosarcoma were included. All 200 HE slides were converted to full digital scanning section WSI by Hamamatsu Optics' NanoZoomer 2.0HT digital section scanner (the files were stored in their proprietary nanozoomer digital pathology image (NDPI) format). The scanning magnification was 20× objective (384‐810 MB) and 40× objective (1.24‐2.86 GB).

### 2.2 Realization and evaluation method of RHR images

With the development of AI, deep learning with deep convolutional neural networks (CNNs)^11^ has been shown to be a powerful algorithm for advancing biomedical image analysis.^12^, ^13^ By studying the development and progress in the field of single image super‐resolution (SISR), we have noted that in the past 2 years, deep learning methods were superior to the traditional methods in terms of the peak signal‐to‐noise ratio (PSNR) and structural similarity (SSIM). Our aim is to zoom in to × 40 from × 20, to adopt a single‐scale model. To further comprehensively consider the effect and computation, we have adopted the champion scheme named Enhanced Deep Super‐Resolution network (EDSR) in the New Trends in Image Restoration and Enhancement 2017 Challenge on SISR.

EDSR is a type of generative network based on sample learning. The general process is to obtain a low‐resolution (LR) image downsampled by interpolation from an HR image as the LR input of a CNN, and the original image is the HR input of a CNN. Subsequently, a large number of such paired samples are used to train the network to establish an end‐to‐end mapping relationship between the LR image and its corresponding HR image. Finally, the mapping relationship is used to create an RHR image from the LR image.

The authors of EDSR have constructed the model baseline with residual blocks, whose structure is similar to that of the SRResNet.^14^ However, the EDSR does not have rectified linear unit activation layers outside the residual blocks. Moreover, the baseline model does not have residual scaling layers and includes only 64 feature maps for each convolution layer.

RHR involves upsampling of the image resolution. The Super‐Resolution Convolutional Neural Network (SRCNN)^15^ applied convolution layers on the pre‐upscaled LR images. This is inefficient because all convolutional layers need to compute in HR space, yielding significantly more computation than in LR space. To accelerate the processing speed without loss of accuracy, the Fast SRCNN (FSRCNN)^16^ utilized a parametric deconvolution layer at the end of the super‐resolution (SR) network, thereby enabling all convolution layers to be computed in the LR feature space. Another non‐parametric efficient alternative is pixel shuffling^17^ (or sub‐pixel convolution). Pixel shuffling is also believed to introduce fewer checkerboard artifacts than the deconvolutional layer.^18^ We have also used pixel shuffling as the upsampling operation.

**FIGURE 1:**
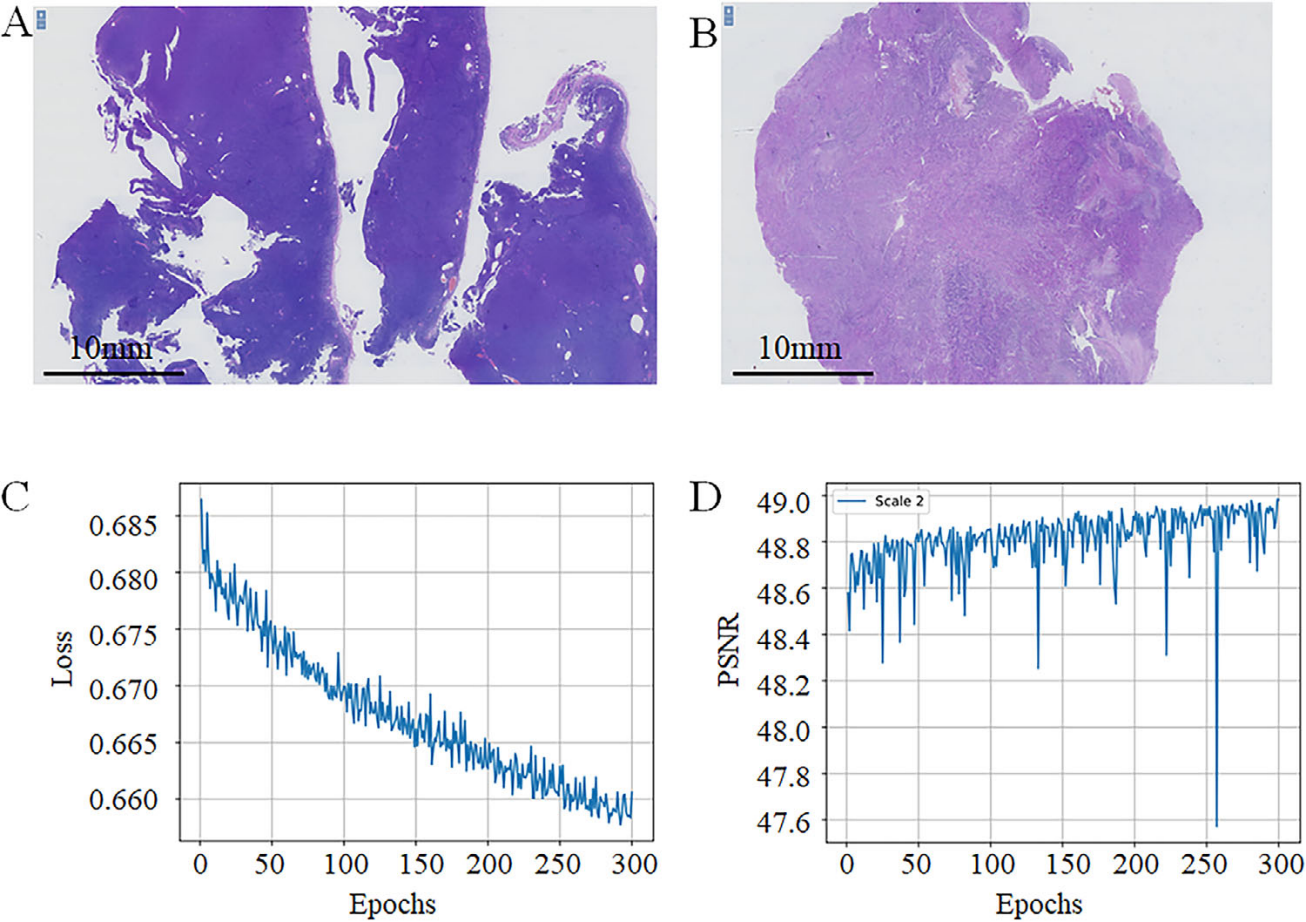
Examples of our datasets. (A), Adult granulosa cell tumor; (B), leiomyosarcoma; (C), training loss curve; (D), curves of PSNR between the RHR and HR images in validating dataset.Abbreviations: HR, high‐resolution; PSNR, peak signal‐to‐noise ratio; RHR, reconstructed high‐resolution. Scale2, twice the magnification

We used the AGCT images and the leiomyosarcoma images as our datasets (Figure 1A,B). We obtained a large number of training patches with a size of 512 × 512 as the HR images randomly split from each training WSI, and validated and tested the patches from each validating WSI and testing WSI, respectively.

Owing to limited computational resources, we only used the baseline model of EDSR. When training, we used RGB input patches of size 256 × 256 from the LR images with the corresponding HR patches. The 512 × 512 RGB input patches from the HR images and their bilinear downsampled images were used as the training output‐input pairs. We preprocessed all images by subtracting the mean RGB value based on the default settings. We also trained our networks using L1 loss instead of L2 because L1 loss can provide better convergence than L2. We used both the GCT and the leiomyosarcoma output‐input pairs. After a few training sessions, the final training loss curve is shown in Figure 1C, and the PSNR curve in the validating dataset is shown in Figure 1D. The last training process used the best model achieved before as the pre‐trained model.

## 3 RESULTS

### 3.1 RHR images quality of the WSI

**FIGURE 2:**
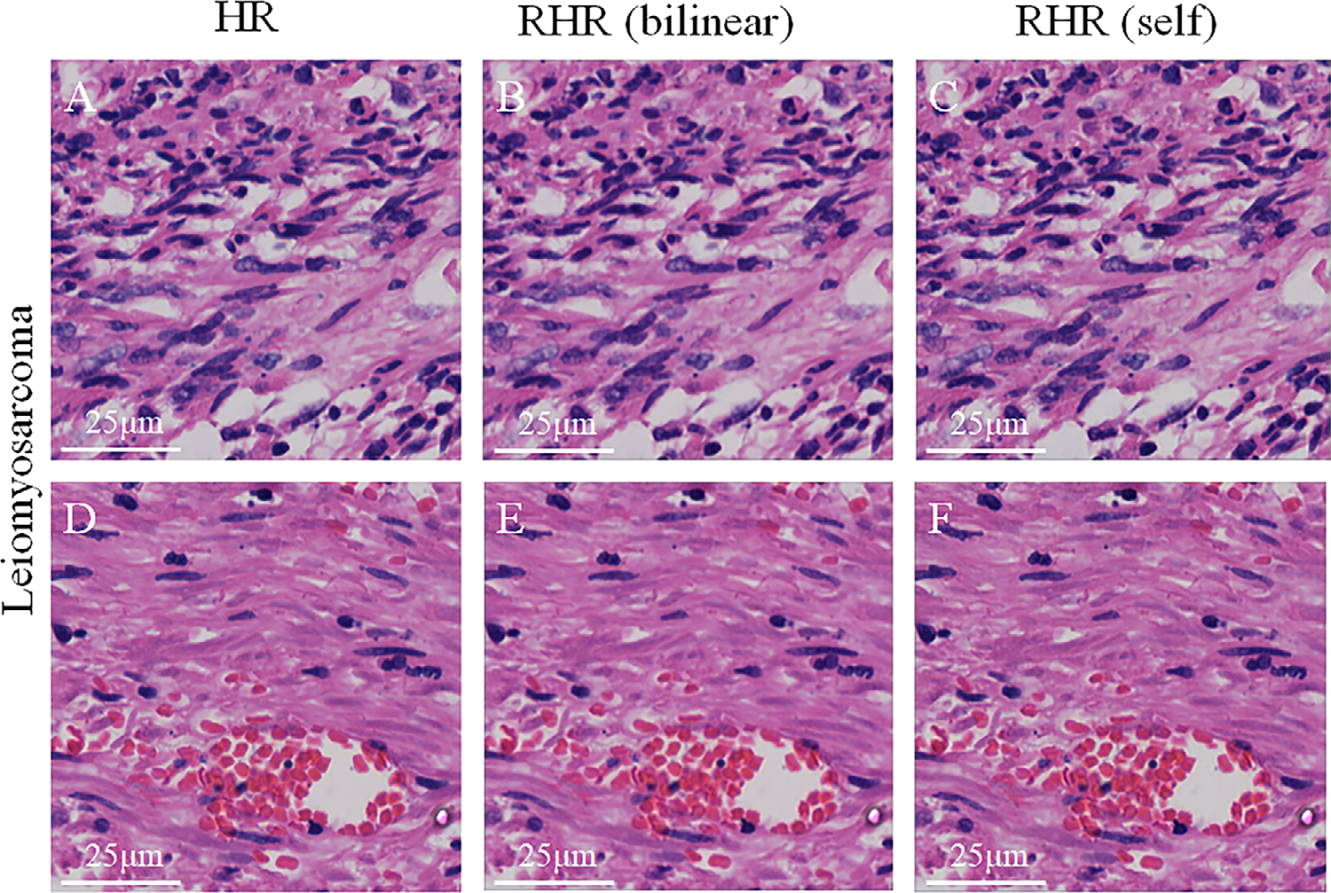
Result comparing between HR and RHR images. All Images are uterine leiomyosarcoma, and the first row and second row are from different samples. (A) and (D) are HR images, (B) and (E) are our RHR images with sr_bilinear_as_lr_input, (C) and (F) are our RHR images with sr_self_as_lr_input.Abbreviations: HR, high‐resolution; RHR, reconstructed high‐resolution; sr_bilinear_as_lr_input, bilinear down‐sampled images from 40 × HR images; sr_self_as_lr_input, images directly from the 20 × images in WSI

We applied our trained model to the testing dataset, including 2000 images with a benchmark. We tested two types of LR inputs with a size of 256 × 256, one included bilinear downsampled images from × 40 HR images (named sr_bilinear_as_lr_input), and the other was directly from the × 20 images in WSI (named sr_self_as_lr_input). The mean PSNR values for RHR and HR images were 43.92 and 42.03, respectively. And the average SSIM values for both RHR and HR images were 0.99. In general, for larger PSNR or SSIM value, the quality of the image is better. The PSNR value assumes infinite value, and the SSIM value is 1.0 when the two compared images are the same. Some image results are shown in Figure 2. We can conclude that our RHR results are extremely similar to the HR images. To a large extent, we have achieved RHR images.

**FIGURE 3:**
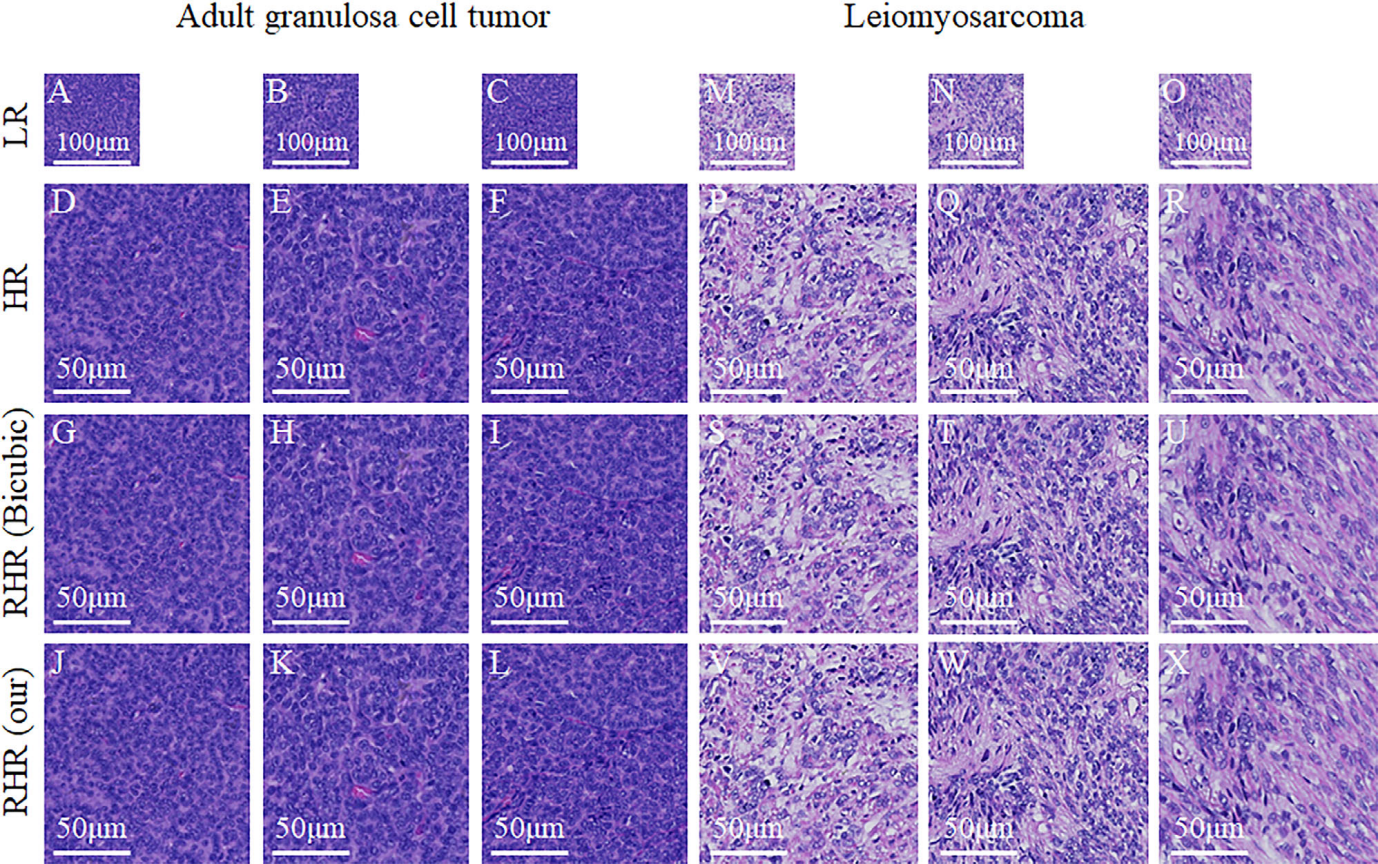
Result samples of AGCT and uterine leiomyosarcoma. The first row (A‐C) and (M‐O) are LR images, the second row (D‐F) and (P‐R) are HR images, the third row (G‐I) and (S‐U) are the RHR images up‐sampled by Bicubic, the last row (J‐L) and (V‐X) are our RHR images. The images (A‐L) on the left are AGCT, and the images (M‐X) on the right are uterine leiomyosarcoma. The first column, second column, and third column are from different samples.Abbreviations: AGCT, adult granulosa cell tumor; HR, high‐resolution; LR, low‐resolution; RHR, reconstructed high‐resolution

We also applied our model to another dataset including 1000 images with an approximate benchmark in which LR images with a size of 256 × 256 were from the true × 20 WSI, and HR images with a size of 512 × 512 were from the other true × 40 WSI. Some image results are shown in Figure 3. The LR images are not downsampled from HR images, and they are from different WSIs. Although we have obtained the similar‐pair images based on the manual registration method up to the maximum possible extent, they do not have one‐to‐one correspondence in pixel‐spatial position. We could not use the PSNR or SSIM metrics to evaluate the results. We used another evaluation method named Blind/Referenceless Image Spatial Quality Evaluator (BRISQUE),^19^ which is a type of no‐reference image quality assessment. Hence, it only needs one image as the input and the image quality score as the output. In general, the score is between zero and 100, and for smaller score, the quality of the image is better. The mean BRISQUE scores of our HR and RHR images and those related to Bicubic on this testing dataset are listed in Table 1. The average calculation time of our model for each image is approximately 2.5 milliseconds on the Graphic Process Unit (GPU). From the results, we can observe that the quality of our results is better than that of the RHR images upsampled by Bicubic, and even better than the HR images. To a large extent, we have achieved RHR images, and our model can be applied to the true × 20 WSI to reconstruct × 40 WSI.

**TABLE 1:** Mean BRISQUE scores of HR images, RHR images up‐sampled by Bicubic and ours

BRISQUE	GT_40 ×	RHR_Bicubic_40 ×	RHR_Ours_40 ×
WSI	52.19	55.90	49.22
Nuclear division	40.97	45.66	39.90
Nuclear groove	49.66	53.61	49.29

Abbreviations: BRISQUE, Blind/Referenceless Image Spatial Quality Evaluator; GT_40 ×, the high‐resolution images; RHR_Bicubic_40 ×, reconstructed high‐resolution images related to Bicubic; RHR_Ours_40 ×, reconstructed high‐resolution images related to ours; WSI, whole slide image.

### 3.2 RHR images quality of the texture details

**FIGURE 4:**
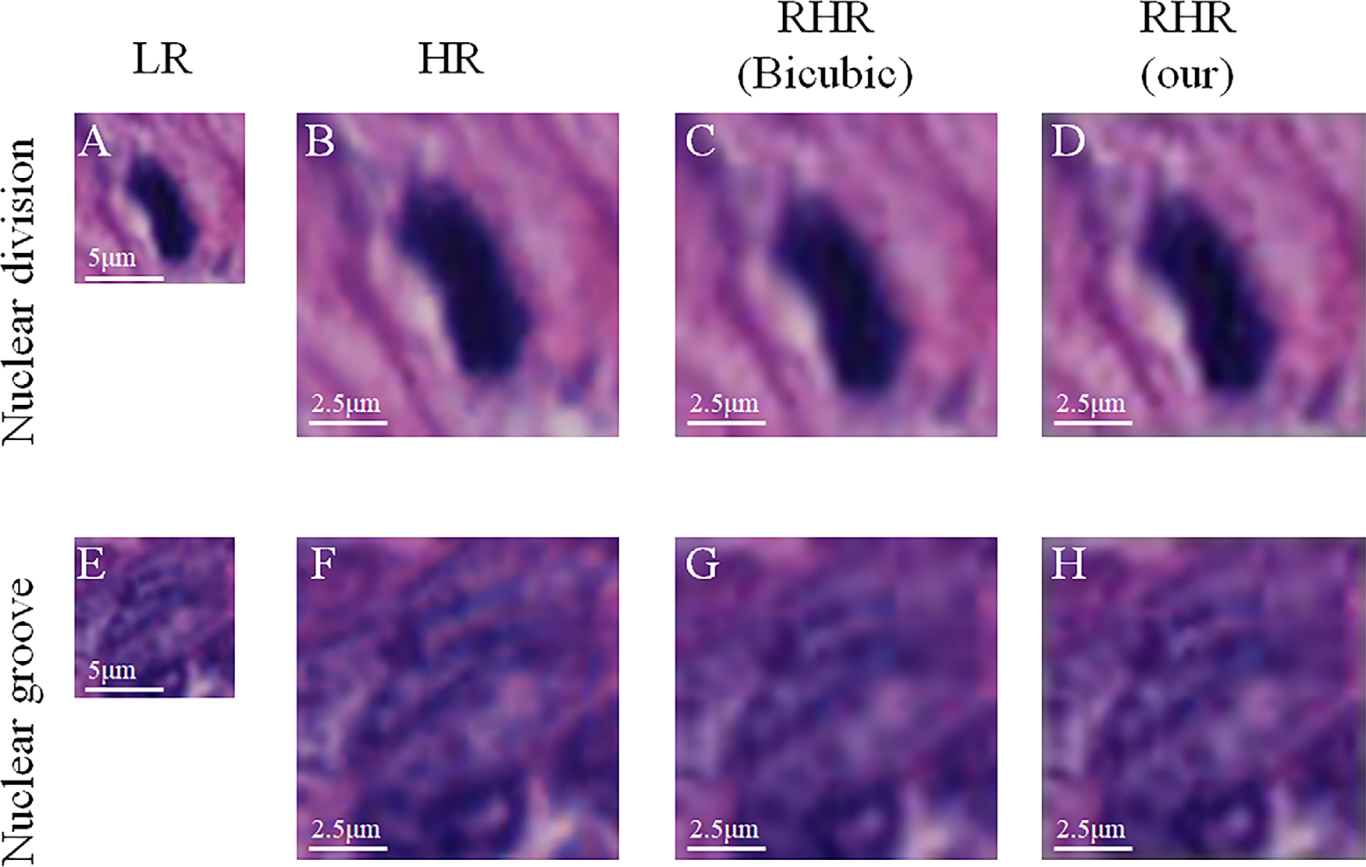
Result samples of nuclear division and nuclear groove. The images (A‐D) are nuclear division images, the images (E‐H) are nuclear groove images; the first column (A and E) is LR images, the second column (B and F) is HR images, the third column (C and G) is the RHR images up‐sampled by Bicubic, and the last column (D and H) is our RHR images.Abbreviations: HR, high‐resolution; LR, low‐resolution; RHR, reconstructed high‐resolution

To further observe the texture details of RHR images, we created two new test datasets, including 192 nuclear division images and 546 nuclear groove images with a size of 32 × 32, which were annotated by doctors. Some image results are shown in Figure 4, and the mean BRISQUE scores of our HR and RHR images and those achieved by Bicubic are listed in Table 1. From these results as well, we can observe that the quality of our results is better than that of the RHR images upsampled by Bicubic, and even better than the HR images. To a large extent, our results can be used directly for subsequent tasks in these datasets.

### 3.3 Pathologist's evaluation results of the RHR images

We then tested the subjective evaluation of our RHR images from the perspective of the pathologist. We selected 1000 images from each uterine leiomyosarcoma and AGCT of the ovary, and provided HR images and our RHR images, respectively. Two pathologists (intermediate titles) evaluated the authenticity of the RHR images subjectively, and considered whether the qualities of the RHR images are adequate for routine usage (diagnostic confidence). The results are shown in Table 2. In addition, we extracted 200 images from each of the two tumors, provided HR images and our RHR images, but without any tags or comments, and allowed the pathologist to determine the true images (HR images). The test results showed that the probability of the two pathologists accurately selecting the actual images was 51.75% and 54.25%, respectively (Table 3). This objectively proves that the pathologists could not easily distinguish the difference between these two types of images and further confirmed the consistency between our RHR images and the actual HR images.

**TABLE 2:** The subjective evaluations of the RHR images from the pathologists

	Pathologist A		Pathologist B	
Opinion	UT	AGCT	UT	AGCT
RHR images’ authenticity rate	95.9%	98.8%	97.5%	91.4%
RHR images' authenticity rate (average)	97.35%	94.45%		
Confidence rate	95.1%	98.8%	97.7%	92.5%
Confidence rate (average)	96.95%		95.1%	

In this table, “RHR images authenticity rate” is equal to the number of no marked difference^*^ pictures divided by the total number of pictures, and “Confidence” means that the pathologist is confident to make a correct diagnosis based on RHR images.

Abbreviations: AGCT, adult granulosa cell tumor; RHR: reconstructed high‐resolution; UL, uterine leiomyosarcoma.

^*^ There is a marked difference, meaning the pathologists think the two kinds of images are so different that they might have affected their diagnosis.

**TABLE 3:** The objective test of the RHR images from the pathologists

	Pathologist A		Pathologist B	
Results of test	UT	AGCT	UT	AGCT
Correct	97	110	105	112
Incorrect	103	90	95	88
Accuracy rate^*^	48.5%	55.0%	52.5%	56.0%
Accuracy rate (average)	51.75%		54.25%	

In this table, “Correct” represents how many high‐resolution images are selected correctly, while “Incorrect” is the opposite, and “Accuracy rate” is equal to the number of correct images divided by the total number of images.

Abbreviations: AGCT, adult granulosa cell tumor; RHR, reconstructed high‐resolution; UL, uterine leiomyosarcoma.

^*^ The value of accuracy is not the higher the better. It represents the probability that the pathologist can accurately distinguish the two kinds of images. The closer the value is to 50%, the more the pathologist cannot distinguish the difference between the two images, and the more our expectation is met.

## 4 DISCUSSION

The testing results indicate that the quality of our RHR images reconstructed from true LR images is better than the RHR images upsampled by Bicubic, and even better than the HR images. This is irrespective of the perspective of vision or evaluation values, and the × 40 WSI synthesized by the RHR process matches the performance of that generated from the × 40 objective in the diagnosis of both tumors. Based on this, we believe that the RHR technology is a reliable method for paleopathological consultation. This can help improve the diagnostic accuracy, reduce time and storage cost, and play an irreplaceable role in treating diseases that must be diagnosed at high magnification.

However, in this article, the RHR technology still has some limitations. First, we only performed a double zoom. If the magnification factor is larger, such as × 4 or × 8, the restoration of the details is not as good. Second, we applied the baseline model to our task because of limited computing resources. Finally, the trained model only applies to uterine leiomyosarcoma and AGCT of the ovary, and we need more data for deep learning to apply this model to other diseases, which might be hampered by a lackno of adequate raw images. Transfer learning can solve this problem well, but it still requires a certain amount of data, hence this method may not be suitable for rare cases in which it is difficult to obtain raw images.

Interestingly, the RHR algorithms are also improving. The authors^14^ of the EDSR proved experimentally that the processing effect can be improved if the network is widened and deepened. Some research^20^ on fractional magnification has been conducted, and it has performed very well. We believe that methods based on deep learning will be increasingly applicable to real tasks. These should be reliable and could be used in the digital slides of a variety of tumors, and can be available on a large scale in clinical pathology as innovative techniques.

## CONFLICT OF INTEREST

The authors declare that there is no conflict of interest that could be perceived as prejudicing the impartiality of the research reported.

### ETHICS APPROVAL AND CONSENT TO PARTICIPATE

Data used in this study were collected as part of medical records. Institutional and national research ethic committee has approved the data collection and management process.

### AUTHOR CONTRIBUTIONS

Ji Bao and Hong Bu designed the study. Yang Deng and Min Feng collected and analyzed all the data. Fei Xiang and Yizhe Wang provided the structure of reconstructed high‐resolution AI models. Yang Deng and Yizhe Wang trained the reconstructed high‐resolution AI models. Min Feng and Yong Jiang tested the model and gave there diagnosis from a doctor's point of view. Yang Deng and Hangyu Qing analyzed the results and optimized the reconstructed high‐resolution AI models accordingly. Yang Deng and Min Feng wrote the manuscript.

### FUNDING INFORMATION

National Key Research and Development Program. Grant number: 2017YFC0113908; Technological Innovation Project of Chengdu New Industrial Technology Research Institute. Grant number: 2017‐CY02‐00026‐GX; 135 project for disciplines of excellence, West China Hospital. Grant number: ZYGD18012; Sichuan International Science and Technology Cooperation and Exchange Research and Development Project. Grant number: 2017HH0070 and 2018HH0037

## Data Availability

The datasets used during the current study are available from the corresponding author on reasonable request.
